# Analysis of Causal Factors to Acute Malnutrition Among Children Under the Age of Five in West Nile, Uganda

**DOI:** 10.1002/fsn3.71684

**Published:** 2026-03-23

**Authors:** Koichiro Watanabe, Shizuka Hori, Catherine Mshilla

**Affiliations:** ^1^ Network for Action Against Malnutrition (NAM) Nakano‐ku Tokyo Japan; ^2^ PACHEDO Foundation Gulu Uganda

**Keywords:** child malnutrition, dietary intake, structural equation modeling, wasting

## Abstract

Addressing child malnutrition requires a comprehensive understanding of its determinants. However, empirical evidence examining the relationships between these determinants and child malnutrition outcomes remains limited in Uganda. This study examined the underlying causes of acute malnutrition among children under 5 years of age in refugee settlements and surrounding host communities in West Nile, Uganda. Quantitative data were collected through a cross‐sectional survey using a two‐stage cluster sampling method including 767 mothers and 1167 children under the age of 5 years. The data collected included household food security, dietary intake, access to health and nutritional services, and anthropometric measurements. Confirmatory factor analysis was used to assess the fit of the hypothetical model, and structural equation modeling (SEM) examined the relationships between latent variables and weight‐for‐height *Z*‐scores. The prevalence of Global Acute Malnutrition was estimated to be 8.8%, and a low proportion of children (7.2%) and their mothers (15.0%) met the minimum dietary diversity requirements. SEM revealed that household food security (*p* = 0.006) and maternal body mass index (*p* < 0.001) had significant positive effects on children's nutritional status, whereas the direct effects of the other factors were not statistically significant. The analysis also revealed that access to health and nutrition services had significant positive effects on dietary intake (*p* = 0.002) and disease incidence (*p* < 0.001). Additionally, dietary intake had a significant protective effect against disease (*p* < 0.001), and, in turn, household food security positively and significantly influenced dietary intake (*p* = 0.042). These findings suggest that future interventions aimed at reducing undernutrition in children, particularly in the context of protracted emergencies, should integrate food security, maternal health, and food delivery services.

## Graphical abstract

.The study examined the underlying causes of acute malnutrition among children in refugee settlements and host communities in West Nile, Uganda. Household food security and maternal body mass index had positive effects on children's nutritional status. Also, access to health and nutrition services had positive effects on diet intake and disease incidence. Diet intake had a protective effect against disease, and, in turn, household food security positively influenced diet intake.

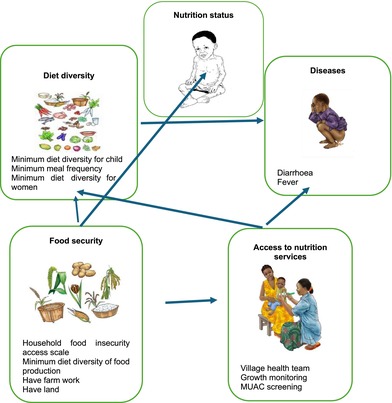

## 1 Introduction

Acute malnutrition is one of the main causes of morbidity and mortality among children under 5 years of age worldwide. In contrast to the prevalence of stunting, which has steadily declined globally over the past two decades from 33.0% in 2000 to 22.3% in 2022, the prevalence of acute malnutrition has remained relatively high (8.7% in 2000 and 6.8% in 2022). For 2022, it was predicted that over 45 million children under 5 years of age would be wasted, including nearly 13.7 million who would be severely wasted. Africa alone accounts for more than 12.2 million wasted children, with the burden disproportionately concentrated in low‐income settings (World Health Organization 2023). Undernutrition remains a major contributor to child mortality, accounting for more than one in three deaths among children under five (Liu et al. 2012). A recent systematic review identified the most consistent factors associated with child malnutrition as maternal education, household income, maternal nutritional status, child age, availability of sanitation facilities at home, family size, birth order, and birth weight (Katoch 2022).

The negative effects of malnutrition on child survival have been well‐documented. For example, underweight children have an ∼2‐ to 8‐fold higher risk of death than those who are adequately nourished (Black et al. 2003). Poor nutrition during childhood, particularly during the first 1000 days from conception, contributes both directly and indirectly to excessive child mortality and morbidity, and has long‐term impacts on cognitive development (Black et al. 2013; Victora et al. 2008).

These nutritional challenges are magnified in fragile settings where health system governance, community stability, and health facilities with available resources may be absent. Fragile settings typically have higher rates of children experiencing wasting, underweight, and stunting. Of the 10 countries ranked as the most fragile, five were also among those with the highest hunger index scores (von Grebmer et al. 2023). In recent years, the world has witnessed a dramatic increase in protracted conflict and displacement. In protracted crises, the factors responsible for malnutrition are exacerbated by disrupted access to food and weakened healthcare systems, resulting in a higher‐than‐average prevalence of both acute and chronic malnutrition.

Uganda has been a refugee‐hosting nation for nearly seven decades. As of April 2021, Uganda hosted the world's third largest refugee population. Most of the 1,482,101 refugees and asylum seekers were women and children. Uganda is also an East African country with high levels of undernutrition, with approximately 23% of children under the age of five having stunted growth and approximately 3.6% being wasted (World Health Organization 2023). West Nile region, which hosts several protracted refugee settlements, including Adjumani, was particularly affected. The 2020 Food Security and Nutrition Assessment (FSNA) Survey indicated that the global acute malnutrition (GAM) rate in the Adjumani refugee settlement was 8.3% and was categorized as “poor” according to the World Health Organization (WHO) standards (Ministry of Health [MoH] et al. 2020).

Addressing the burden of childhood malnutrition requires a comprehensive understanding of its determinants within specific contexts, including individual, household, community, regional, and environmental levels (Black et al. 2020). The framework conceptualized by the United Nations International Children's Emergency Fund (UNICEF) identifies three categories of malnutrition determinants: basic, underlying, and immediate (UNICEF 1990). However, limited evidence is available on the causal factors of acute malnutrition in low‐income countries and refugee‐hosting regions, such as West Nile, Uganda. A pooled analysis of data collected in Haiti, Burkina Faso, and Madagascar reported that being male, a history of diarrhea, and having an unwashed face and hands were significantly associated with GAM as defined by the WHZ (Nassur et al. 2022). Another cross‐sectional survey in Ethiopia found that family size, younger age, and a history of diarrhea in the 2 weeks prior to the survey were independently associated with severe acute malnutrition in the study population (Anato 2022). Evidence of the causal factors of acute malnutrition is limited in Uganda. Analysis of large‐scale nutrition surveys conducted in Karamoja found that concurrent wasting and growth stunting among children was associated with being male, having acute respiratory infections, episodes of diarrhea, maternal undernutrition, high parity (≥ 4 live births), and living in households without livestock (Odei Obeng‐Amoako et al. 2021).

Government efforts to address acute malnutrition have largely focused on curative services through the Integrated Management of Acute Malnutrition program. With the current trend of reduced international aid globally and in West Nile, effective preventive strategies that promote self‐reliance and mitigate the negative impacts of reduced aid are increasingly needed. Formulating these strategies requires an in‐depth understanding of the causal factors. Therefore, this study examined the underlying causes of acute malnutrition in the refugee settlements of West Nile and the surrounding host communities. The findings will inform the government of Uganda and its partners about the efficacy of the program and inform evidence‐based learning and planning.

## 2 Materials and Methods

### 2.1 Study Design, Period, and Setting

This study analyzed data collected from a baseline survey completed between January and February 2024 including children aged 0–59 months and their caregivers. The baseline survey employed a cross‐sectional design using a two‐stage cluster sampling method. Data were collected from the Ayilo I and II Refugee Settlements and the surrounding host communities (Pakele and Dzaipi) in the Adjumani District, West Nile, Uganda. The Nutrition Improvement and Livelihood Empowerment (NILE) project is being implemented in these areas by the Network for Action against Malnutrition in partnership with the Partners for Community Health and Development Organization (PACHEDO), with the aim of reducing malnutrition among pregnant and lactating women, adolescents (12–19 years), and children under five and to improve the livelihoods of the targeted households over a 3‐year period, from November 2023 to October 2026.

### 2.2 Study Participants and Sampling Procedure

The baseline survey was designed to assess the nutritional status and factors contributing to undernutrition among children aged 6–59 months as part of a three‐arm randomized cluster trial for an impact study. Based on the average number of eligible children aged 0–59 months and women of reproductive age (1.6 per household) derived from previously conducted surveys in the district, the total sample size was estimated to be 1000 households. With a fixed number of clusters (50) and a fixed number of households per cluster (20), and assuming a two‐sided significance level of 0.05 and a desired power of 80%, the sample size was deemed sufficient to detect a 6–13 percentage point difference in key indicators. This calculation was based on an intraclass correlation coefficient of 0.25 to account for clustering effects.

Clusters were defined as blocks in the settlements and villages of the host communities. There are 51 blocks within the Ayilo I and II refugee settlements and 61 villages in the host communities. After excluding the clusters that received special programs or assistance, 50 clusters were selected using probability proportional‐to‐size sampling. Within each selected cluster, the data collectors conducted a complete listing of households with children aged 0–59 months. Twenty households were selected from this list using systematic random sampling. All eligible children within the selected households and their mothers were included in the study.

The inclusion criteria were willingness to participate and having a child aged 0–59 months, whereas unwillingness to participate or having no children aged 0–59 months were applied as the exclusion criteria.

### 2.3 Data Collection Methods

The questionnaire comprised eight modules. The household‐level modules included basic characteristics, household food security, dietary diversity, agricultural practices (including food production and consumption), and women's empowerment. A module on health and nutrition knowledge and practices was administered to caregivers of children aged 0–23 months. Another module, targeting households with children aged 0–59 months, focused on primary health and nutrition services and was directed at the mother of the child. The final module focused on anthropometry in children aged 6–59 months. All household survey questionnaires were designed for face‐to‐face interviews and were field‐tested prior to deployment.

Anthropometric measurements of children aged 6–59 months included age, sex, weight, and height/length to assess nutritional status by age and sex. Body weight was measured using standard techniques with a mother and child electronic scale and recorded to the nearest 0.1 kg. Length or height was measured in either the lying or standing position using standard techniques, and infant/child/adult height boards were recorded to the nearest 1 mm. Standardized anthropometric skills were taught during survey team training to minimize inter‐observer measurement bias.

Data quality control included comprehensive training of data collectors to ensure an in‐depth understanding of the study protocol, analytical framework, and standardized procedures for effective quantitative and qualitative data collection. Digital tools were used for real‐time data entry and quality checks.

### 2.4 Definition of Childhood Growth Indices

Wasting in children was defined using weight‐for‐height index values and classified according to the WHO Child Growth Standards. WHZ scores < 6 or > 6 were excluded for biological implausibility, in line with the WHO guidelines.

### 2.5 Factor Variables

A range of factors potentially associated with acute malnutrition in children aged 6–59 months was examined in this analysis, informed by previous literature and the UNICEF conceptual framework for undernutrition (UNICEF 1990).

Dietary intake: Each child's mother was asked about the foods and beverages that she and her child consumed in the past 24 h. The minimum dietary diversity of children (MDD‐C) was coded as 1 if a child aged 6–23 months consumed food from at least five of the eight defined food groups. The minimum dietary diversity of women (MDD‐W) was coded as 1 if a woman consumed foods from at least five of the defined food groups. Minimum meal frequency (MMF) was coded as 1 if breastfed and non‐breastfed children aged 6–23 months received solid, semi‐solid, or soft foods (including milk feeds for non‐breastfed children) a minimum number of times or more.

Disease experience: Diarrhea, fever, and cough in the past 2 weeks (yes/no) were included in the questionnaire.

Household food security: The food security status of households was determined based on the Household Food Insecurity Access Scale (HFIAS) and Food Consumption Score (FCS).

Access to health/nutrition services: The data included participation in women's groups, Vitamin A supplementation, growth monitoring, mid‐upper arm circumference (MUAC) screening, home visits by village health teams, and iron‐folic acid supplementation.

Maternal age was dichotomized as above or below 40 years. Education level was categorized as no education or at least 1 year of education. The number of children under the age of five per caregiver was classified as one or more than two. Maternal body mass index (BMI) was used as a continuous variable.

### 2.6 Data Analysis

Descriptive statistics were used to summarize the demographic characteristics of the caregivers who participated in the survey and potential factors associated with acute malnutrition. The Average Variance Extracted (AVE) and Composite Reliability (CR) were calculated to assess convergent validity. To evaluate discriminant validity, the square roots of the AVE values and the absolute values of the correlation coefficients between the constructs were examined. Confirmatory factor analysis (CFA) was conducted to assess whether the hypothetical model had a good fit. Structural equation modeling (SEM) was applied to examine the relationships between latent variables, weight‐for‐height *Z*‐scores, and BMI. All statistical analyses were performed using R software. Statistical significance was set at *p* < 0.05. Diagonally Weighted Least Squares (DWLS) was used to estimate the model parameters. Pairwise deletion was used to estimate the models when there were missing values. The minimum sample size recommended for SEM research using the DWLS estimator for binary data was 200–500 (Bandalos 2014); and the number of eligible respondents in this study (*N* = 425) met this requirement.

## 3 Results

### 3.1 Characteristics of the Study Participants

The survey included 767 mothers and 1167 children under the age of 5 in two refugee settlements (79%) and host communities (21%). Mothers aged 30–39 years constituted the largest group (39.9%), followed by those aged 20–29 years (31.4%), those over 40 years (24.1%), and adolescents (10–19 years, 5.1%). The majority of mothers (86%–93%) were married, and around four in every 10 (36.9%) did not have any formal education. The major religion was Catholicism (88.6%), and the main ethnic group was Dinka (69%) (Table 1).

**TABLE 1:** Characteristics of study participants.

Variable	Frequency (*n*)	Percentage (%)
*Child age in months*		
0–11	170	14.60%
12–23	242	20.70%
24–35	233	20.00%
36–47	260	22.30%
48–59	262	22.50%
*Gender of child*		
Female	471	46.1%
Male	551	53.9%
*Residence*		
Host community	215	21.0%
Refugee settlement	807	79.0%
*Number of child less than 5 years*		
One	418	40.9%
More than2	604	59.1%
*Ethnicity*		
Dinka	707	69.2%
Others	315	30.8%
*Religion*		
Catholic	424	41.5%
Others	598	58.5%
*Maternal age less than 20*		
20 years or above	981	96.1%
Less than 20	40	0.90%
*Education level of mother*		
No education	368	36.9%
At least 1 year	630	63.1%
*Marital status of mother*		
Married	894	87.5%
Others	128	12.5%

### 3.2 Prevalence of Malnutrition

The prevalence of GAM is estimated to be 8.8%, with approximately 2% classified as severely wasted. The overall mean (±SD) of the weight‐for‐height/length *Z*‐score was −0.565 ± 1.24, with no significant difference between boys and girls (*p* = 0.888). The mean (±SD) BMI of mothers was 20.7 ± 3.13, and 23.8% of mothers were classified as underweight. The proportion of wasted children declined from 10.2% among those aged 6–17 months to 6.7% among those aged 18–29 months, reaching the lowest level of 4.4% in children aged 30–41 months, and then gradually increased to 12.2% in children aged 42–59 months. The prevalence of wasted children was higher in boys (9.9%) than in girls (7.5%) (Table 2).

**TABLE 2:** Nutritional status of children and mothers.

Indicators	Child age (in months)				Child gender		Total
	6–17	18–29	30–41	42–0.59	Female	Male	
Child acute malnutrition^a^							
Moderate, *n* (%)	18 (8.7%)	9 (3.8%)	6 (2.6%)	37 (10.3%)	29 (6.0%)	41 (7.4%)	70 (6.8%)
Severe, *n* (%)	3 (1.5%)	7 (2.9%)	4 (1.7%)	7 (1.9%)	7 (1.5%)	14 (2.5%)	21 (2.0%)
Global, *n* (%)	21 (10.2%)	16 (6.7%)	10 (4.4%)	44 (12.2%)	36 (7.5%)	55 (9.9%)	91 (8.8%)
WHZ, mean (SD)	−0.29 (1.5)	−0.55 (1.1)	−0.49 (1.1)	−0.78 (1.2)	−0.55 (1.2)	−0.58 (1.3)	−0.57 (1.2)
Maternal undernutrition^b^							
Underweight, *n* (%)							213 (23.8%)
Normal, *n* (%)							599 (66.9%)
Overweight, *n* (%)							77 (8.6%)
Obesity, *n* (%)							6 (0.7%)
BMI, mean (SD)							20.7 (3.1)

Abbreviations: BMI, body mass index; WHZ, weight‐for‐height *Z*‐score.

^a^ Child acute malnutrition—defined using WHZ: for severe, < −3; for moderate, between −3 and −2; for global, < −2.

^b^ Maternal undernutrition—defined using BMI; for underweight, < 18.5; for normal, between 18.5 and 24.9; for overweight, between 24.9 and 30; for obesity, > 30.

### 3.3 Potential Factors to Malnutrition

Infant and young child feeding practices were suboptimal, as illustrated by the exclusive breastfeeding rates of 64%, minimum dietary diversity of 4.7%, and MMF of 20.4% among children 6–23 months. The survey found that almost one in every four children (23.6%) aged 0–59 months had a diarrheal episode in the 2 weeks preceding data collection, whereas fever and cough were reported in nearly half of the children (51.4% and 45.5%, respectively). Access to basic health and nutrition services was low: 74.0% of children had received vitamin A supplementation in the last 6 months, whereas growth monitoring and promotion and MUAC screening coverage were 34.8% and 44.9%, respectively. The survey found that only a small proportion of women (14.7%) consumed more than five food groups (MDD‐W). Although the vast majority (97%) of households had access to safe water sources, only a small proportion (6.4%) had handwashing stations where water and soap were available. Low levels of household food security were indicated by a high proportion of households with insufficient FCS (poor and borderline, 72.4%) and severe HFIAS (59%–66%). Of the women, 76.7% reported participating in all decisions with their husbands, either independently or jointly. Overall, 39.3% of the women reported that their husbands supported childcare (Table 3).

**TABLE 3:** Potential factors to child malnutrition.

Variables	*N* (%)		*p*
	Female	Male	
Infant and young child feeding practices			
Minimum diet diversity of children	10 (7.2%)	9 (5.4%)	0.506
Minimum meal frequency	31 (22.3%)	29 (17.3%)	0.268
Minimum diet diversity of women	72 (15.0%)	83 (15.0%)	0.986
Early initiation of breastfeeding within 1 h after birth	113 (85.0%)	134 (84.8%)	0.971
Continued breastfeeding for 12 months or beyond	71 (77.2%)	87 (75.7%)	0.798
Use bottle for feeding child	11 (7.9%)	13 (7.7%)	0.955
Use milk for child feeding	54 (38.8%)	52 (31.0%)	0.147
Disease experiences			
Child experienced diarrhea in last 2 weeks	127 (26.4%)	136 (24.6%)	0.505
Child experienced fever in last 2 weeks	266 (55.3%)	313 (56.6%)	0.675
Child experienced cough in last 2 weeks	242 (50.3%)	272 (49.2%)	0.718
Access to basic health and nutrition services			
Mother participated women group in last 60 days	116 (24.1%)	133 (24.1%)	0.98
Mother received Vitamin A supplementation in last 60 days	366 (76.1%)	408 (73.8%)	0.393
Mother received GMP in last 30 days	190 (39.5%)	197 (35.6%)	0.199
Mother received MUAC screening in last 6 months	242 (50.3%)	258 (46.7%)	0.24
Mother received VHT home visit in last 3 months	171 (35.6%)	181 (32.7%)	0.34
Mother received IFA supplementation during pregnancy	396 (82.3%)	452 (81.7%)	0.805
Household food security			
Household food insecurity access (moderate)	42 (8.7%)	39 (7.1%)	0.316
Household food insecurity access (severe)	432 (89.8%)	507 (91.7%)	0.299
Food consumption score (borderline)	178 (37.0%)	184 (33.3%)	0.209
Food consumption score (poor)	168 (34.9%)	222 (40.1%)	0.0843
Food production diversity (> 5 food groups produced)	79 (16.4%)	88 (15.9%)	0.824
Have income	310 (64.4%)	326 (59.0%)	0.0699
Have land	168 (34.9%)	173 (31.3%)	0.214
Have farm work	258 (53.6%)	280 (50.6%)	0.335
Water, sanitation, and hygiene			
Access to safe water	471 (97.9%)	535 (96.7%)	0.245
Treat water to drink	37 (7.7%)	46 (8.3%)	0.712
Have handwashing station with water and soap	25 (5.2%)	32 (5.8%)	0.679
Have toilet at household	277 (57.6%)	327 (59.1%)	0.615
Keep animals in confined space	47 (9.8%)	56 (10.1%)	0.849
Keep poultry in cage	74 (15.4%)	85 (15.4%)	0.995
Gender			
Husband support for child care	194 (40.3%)	209 (37.8%)	0.404
Participation of women in household decision‐making	319 (66.3%)	385 (69.6%)	0.256

Abbreviations: GMP, growth monitoring promotion; IFA, iron‐folic acid; MUAC, mid‐upper arm circumference; VHT, village health team.

### 3.4 SEM

#### 3.4.1 Discriminant Validity Analysis and Testing the Fit of the Model

The Kaiser–Meyer–Olkin (KMO) measure of sampling adequacy and Bartlett's test of sphericity confirmed that the respondents' data were suitable for factor analysis. The initial KMO measure for the 27 variables identified 9 items with low KMO values (< 0.5), which were excluded. The remaining 18 items had a KMO value of 0.67, with all individual items having a KMO value > 0.5. Bartlett's test of sphericity remained significant (*p* < 0.05), thus meeting the criteria for factor analysis.

Exploratory factor analysis examined the factor structure of all 18 items, refined the item set, and identified four constructs: (1) access to health/nutrition services, (2) household food security, (3) disease experience, and (4) dietary intake. CFA, using DWLS estimation, confirmed that the final constructs consisted of 12 items with factor loadings ranging from 0.500 to 1.077. The acceptable values for CR and AVE were 0.70 and 0.50, respectively. All constructs met these criteria except for disease experience, whose AVE and CR fell outside the acceptable range (Table 4).

**TABLE 4:** Confirmatory factor loadings of the indicators.

Indicators	Factor loadings	AVE	CR
Access to health and nutrition services			
Growth monitoring promotion	0.792	0.772	0.892
MUAC screening	1.077		
Home visits by Village Health Teams	0.728		
Household food security			
Have farm work	0.885	0.694	0.893
Have land for production	0.933		
Food production diversity	0.770		
Household food insecurity^a^	0.728		
Disease			
Child experienced diarrhea in the last 2 weeks	0.655	0.359	0.521
Child experienced fever in the last 2 weeks	0.538		
Dietary intake			
Minimum diet diversity of child	1.074	0.589	0.727
Minimum meal frequency	0.500		
Minimum diet diversity of women	0.607		

Abbreviations: AVE, average variance extracted; CR, composite reliability.

^a^ Household food insecurity—defined using Household Food Insecurity Access Scale (HFIAS) either severe or moderate.

The CFA model's goodness‐of‐fit parameters met the recommended criteria. Although the chi‐square test was significant (*p* < 0.001), the Comparative Fit Index (CFI = 0.982) and Tucker–Lewis index (TLI = 0.974) were > 0.90, showing a good model fit. The Root Mean Square Error of Approximation (RMSEA) was 0.031, which was below the suggested cutoff of 0.08. Additionally, the square roots of the AVE values were higher than the absolute values of the correlation coefficients among attitudes, perceived benefits, and perceived barriers, indicating that the questionnaire had good discriminant validity (Table 5).

**TABLE 5:** Correlation matrix of the four constructs.

	Access to health/nutrition services	Household food security	Disease experience	Diet intake
Access to health/nutrition services	**0.879**			
Household food security	0.179***	**0.833**		
Disease experience	0.129***	0.098***	**0.599**	
Diet intake	0.210***	0.103	0.246***	**0.767**

*Note:* Bold values indicate the square root of the AVE value.

*** *p* < 0.01.

#### 3.4.2 Structural Equation Modeling Fitting for IYCF Behaviors

**FIGURE 1:**
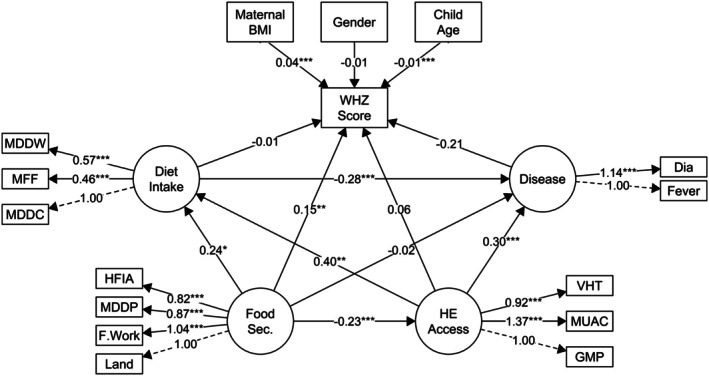
The structural equation modeling for causal factors to acute malnutrition. Circles in the SEM diagram indicate latent variables, whereas rectangles represent observed or measured variables. Arrows between circles and rectangles indicate the paths and relationship between variables, as denoted by their coefficients. Dia, have diarrhea; F. work, have farm work; Fever, have fever; GMP, received growth monitoring; HFIA, household food insecurity access scale (medium/acceptable); MDDP, minimum diet diversity of production; Land, have land; MDDW, minimum diet diversity of women; MFF, minimum feeding frequency; MDDC, minimum diet diversity of child; MUAC, received MUAC screening; VHT, received visit of Village Health Team.

This study aimed to assess whether access to health/nutrition services, household food security, disease experience, and dietary intake affect children's nutritional status. Figure 1 shows the final structural model, which incorporates adjustments based on modification indices to enhance the model fit. Table 6 provides insight into the direct effects of the model. Although the chi‐square test for the model was significant, alternative fit indices indicated a good fit to the data, with a CFI of 0.983 and an RMSEA of 0.027, which was below the suggested cutoff of 0.08. The results indicated that household food security (*β* = 0.147, *p* = 0.006) and maternal BMI (*β* = 0.043, *p* < 0.001) significantly contribute to child nutritional status, as measured by higher weight‐for‐height/length *Z*‐scores. Access to health and nutritional services had significant positive effects on dietary intake (*β* = 0.403, *p* = 0.002) and disease incidence (*β* = 0.302, *p* < 0.001). Additionally, dietary intake had a significant protective effect against diseases (*β* = −0.277, *p* < 0.001) and food security positively and significantly influenced dietary intake (*β* = 0.235, *p* = 0.042).

**TABLE 6:** Path coefficient model of structural equation model.

	Effect paths	Estimate	*T* statistics	p
WFH *z*‐score	Access to service	0.058	0.556	0.578
	Diet intake	−0.009	−0.081	0.935
	Disease	−0.213	−1.009	0.313
	Food security	0.147	2.767	0.006
	Maternal BMI	0.043	3.483	0.000
	Gender	−0.007	−0.091	0.928
	Age	−0.012	−5.312	0.000
Access to service	Food security	−0.225	−5.679	0.000
Disease	Access to service	0.302	4.358	0.000
	Diet intake	−0.277	−3.613	0.000
	Food security	−0.020	−0.414	0.042
Diet intake	Access to service	0.403	3.069	0.002
	Food security	0.235	2.029	0.042

Abbreviation: WFH, weight‐for‐height.

### 3.5 Mediation Analysis

As significant associations were found between access to health/nutrition services, dietary diversity, and disease experience, a mediation analysis was conducted to examine the mediating effect of dietary diversity on access to health/nutrition services and disease experience. The total effect of the model was significant (*β* = 0.190, *p* < 0.001). Both the direct effect (*β* = 0.302, *p* < 0.001) and the indirect effect (*β* = −0.112, *p* = 0.011) were significant. These results suggest that dietary diversity partially mediates the relationship between access to health and nutrition services and disease experience.

## 4 Discussion

This study investigated the prevalence of acute malnutrition among children aged 6–59 months in refugee settlements and surrounding host communities in West Nile and found it to be approximately 8.8%, which closely aligns with the 8.3% reported in the latest FSNA.

SEM was used to investigate the potential causal relationships among various determinants of children's nutritional status. The analysis revealed that household food security (*β* = 0.147, *p* = 0.006) and maternal BMI (*β* = 0.043, *p* < 0.001) significantly contributed to child nutritional status, as measured by higher weight‐for‐height/length *Z*‐scores.

Household food security, as a latent variable encompassing access to land, farm labor, and the diversity of agricultural production, reflects the four pillars of food security: availability, access, utilization, and stability. Despite Uganda's policy of granting refugee households plots for cultivation, food insecurity remains high because of land fragmentation, insecure tenure, and reliance on limited food assistance. These factors impede farmers from investing in sustainable land management practices (Mwesigye and Barungi 2021), thereby limiting the households' capacity to improve nutritional outcomes. This finding is consistent with studies in Ethiopia, Bangladesh, and Malaysia linking household food insecurity to child wasting (Lye et al. 2023; Fufa and Laloto 2021; Choudhury et al. 2017; Motbainor et al. 2015; Eunice et al. 2014).

Household food security also positively predicted dietary intake (*β* = 0.235, *p* = 0.042), underscoring its central role in improving child feeding practices. This finding is consistent with a previous study in Uganda using structural equation modeling, which demonstrated that household‐level dietary diversity is positively associated with crop diversification (Morrissey et al. 2023). Together, these findings highlight the importance of improving access to land and promoting the diversification of food production as strategies for preventing acute malnutrition and enhancing dietary diversity. Although food assistance, both cash and in kind, remains the primary source of food and livelihood for refugees in northern Uganda, recent reductions, coupled with rising food prices, have weakened household purchasing power. Consequently, many households are only able to meet a fraction of their nutritional needs, increasing the risk of further deterioration in food security and nutrition outcomes. These findings highlight the urgent need for effective, preventive, and multisectoral strategies that maximize the use of locally available foods and improve access to nutrient‐dense diets through enhanced farmland access and crop diversification.

Maternal BMI was also positively associated with children's weight‐for‐height *Z*‐scores, providing evidence that maternal nutritional status plays a critical role in child growth. A recent systematic review reported similar evidence, suggesting a possible effect of maternal undernutrition on subsequent child nutritional status (González‐Fernández et al. 2024). Maternal undernutrition, indicated by a low BMI, MUAC, and short stature, has been linked to intrauterine growth retardation, preterm birth, low birth weight, and small‐for‐gestational‐age infants. Studies have examined the association between maternal and child nutritional status, some of which found that maternal BMI is associated with child stunting and underweight (Goudet et al. 2011), as well as with the weight‐for‐height *Z*‐score (Negash et al. 2015).

This study also revealed that access to health/nutrition services had a significant and positive influence on dietary intake (*β* = 0.403, *p* = 0.002), suggesting that adequate and diversified diets depend partly on knowledge and counseling provided through these services. This finding is consistent with existing literature on the determinants of dietary diversity. For instance, a recent study on the determinants of minimum dietary diversity among children aged 6–23 months in three sub‐Saharan African countries reported that those who visited a healthcare facility in the last 12 months were more likely to have MDD (Ba et al. 2022). In Uganda, maternal, infant, young child, and adolescent nutritional messages have been promoted through health facilities and community volunteers. However, the baseline survey in this study revealed persistent gaps in caregivers' knowledge of maternal and child dietary needs, highlighting the importance of further strengthening health and nutrition services as a preventive strategy to improve child feeding practices.

This study also identified a significant role for dietary intake in disease prevention. Children with more diverse diets tended to have better overall food intake, improved metabolism, and were more likely to have good health. Although very few studies have investigated the impact of low food diversity on health status, one study reported a significant negative association between dietary diversity and health complaints, such as flu, cough, and fever (Hasanah et al. 2024), reinforcing the protective role of dietary diversity.

Interestingly, access to health and nutrition services was positively associated with disease experience (*β* = 0.302, *p* < 0.001), a finding that appears counterintuitive. Previous literature suggests that, apart from services such as immunization and mosquito net distribution, the overall impact of health services on population health outcomes, particularly on health equity, has often been unclear, negligible, or even detrimental (Besnier et al. 2019). One plausible explanation for this finding is reverse causality, whereby caregivers are more likely to seek health services when their child is already ill, thereby inflating the observed association. However, mediation analysis indicated that dietary diversity partially mediated the association between access to health and nutrition services and disease experience. Thus, increased access to services improves dietary diversity, which subsequently reduces disease experience.

This study identified several potential causal factors of acute malnutrition and the relationship between them. However, several questions remain unanswered. The results indicated that household food security influences both children's dietary intake and nutritional status. At the same time, as the relationship between dietary intake and nutritional status was not confirmed in the model, the pathway connecting household food security and nutritional status was not fully understood. Findings from other research on the pathways linking dietary diversity and nutritional status have been conflicting. Some studies did not detect a significant association between dietary diversity and nutritional status (Kuchenbecker et al. 2017; Gelli et al. 2018; Osei et al. 2017). Similarly, a meta‐analysis of cross‐sectional studies in Asia concluded that inadequate dietary diversity increases the risk of undernutrition in terms of linear growth, but not thinness. (Zeinalabedini et al. 2023). These contrasting findings suggest that the relationship between dietary diversity and acute malnutrition may be context‐specific and influenced by additional mediating or moderating factors.

Previous studies have also highlighted the contribution of agricultural production to nutrition outcomes through increased income, improved purchasing power, and women's empowerment, which in turn enhance child nutrition (Ruel et al. 2013; Herforth and Harris 2014; Pandey et al. 2016). Although this study collected data on women's income status and empowerment, the measurement approaches were not sufficiently robust to allow clear examination of their mediating roles in the pathways linking household food security and nutritional status. Considerably more research is needed to examine the relationships between agricultural production, nutrient intake, and nutritional status through the potential mediating factors.

Similarly, this study demonstrated a relationship between dietary intake and access to health and nutrition services. However, to gain a more fundamental understanding of the relationship, it is necessary to examine how health and nutrition services influence the knowledge, attitudes, and practices necessary for adequate dietary intake. More information on the behavioral determinants of dietary intake would help establish a clearer and more comprehensive understanding of this matter.

### 4.1 Strength and Limitations

This study examined the causal factors of acute malnutrition in children using a validated model. These findings contribute to a deeper understanding of the complex and multifaceted causes of malnutrition and provide valuable insights into the design and implementation of targeted, sustainable, nutrition‐sensitive, and nutrition‐specific interventions. Despite these strengths, this study has several limitations that should be acknowledged. First, sampling was restricted to selected refugee settlements and their surrounding communities, which limited the generalizability of the findings to other settings. Although an exhaustive listing of all households both in the settlements and host communities was conducted to ensure a comprehensive sampling for households with children under 5 years of age, the high mobility of settlement‐based populations meant that some selected households were unavailable at the time of data collection. This may further limit the representativeness of the sample, particularly for highly mobile populations.

Methodologically, because this was a cross‐sectional study, it could not establish causality. In addition, although the data collectors received thorough training to minimize recall bias, the data relied on caregivers' self‐reports and may therefore be subject to recall and social desirability biases. These design and data collection constraints place greater importance on the quality and robustness of the instruments used in the analysis. This was also evident in the present study. For example, the latent variables initially developed for water, sanitation, hygiene, and women's empowerment did not meet the criteria for reliability and could not be validated. Additionally, the disease experience variable included only two indicators, limiting its robustness. Another source of weakness in this study that could have affected the analysis of causal pathway was that the measurement tool for income was not sufficiently validated prior to data collection. Therefore, more rigorous survey questions and better‐developed measurement instruments are needed to address these limitations and strengthen the quality and validity of future research.

## 5 Conclusions

This study examined the causal factors of acute malnutrition among children aged 6–59 months in West Nile, Uganda and demonstrated the effectiveness of SEM and factor analysis as valid analysis tools. These findings indicate that the prevalence of malnutrition in children and women is unacceptably high. Household food security and maternal BMI were identified as significant positive predictors of child nutritional status, whereas access to health and nutrition services was significantly associated with improved dietary intake among children, suggesting the importance of service‐based nutrition counseling in promoting adequate and diversified diets. The findings of this study have several practical implications for prevention strategies of acute malnutrition in refugee settlements in Uganda. One of the key policy priorities for prevention of acute malnutrition among refugees should be to promote food production diversity through improved access to farmland, alongside targeted support for maternal nutrition. In addition, the results provide valuable insights for the design and implementation of sustainable and targeted nutrition‐sensitive and nutrition‐specific interventions globally. In contexts of protracted emergencies, interventions aimed at reducing child undernutrition should adopt integrated approaches that strengthen household food security, improve maternal nutritional status, and enhance access to effective health and nutrition services.

## Author Contributions

**Catherine Mshilla:** writing – review and editing. **Koichiro Watanabe:** conceptualization, data curation, formal analysis, methodology, investigation, supervision, software, validation, funding acquisition. **Shizuka Hori:** writing – review and editing.

## Funding

The authors have nothing to report.

## Ethics Statement

This study was approved by the Gulu University Research Ethics Committee (reference number: GUREC‐2023‐736). Before data collection, all study staff members completed a research ethics training program. Verbal informed consent was obtained from all participants before the interviews were conducted.

## Conflicts of Interest

The authors declare no conflicts of interest.

## Data Availability

The datasets generated and analyzed in this study are available from the corresponding author upon reasonable request.

## References

[fsn371684-bib-0001] Anato, A. 2022. “Severe Acute Malnutrition and Associated Factors Among Children Under‐Five Years: A Community Based‐Cross Sectional Study in Ethiopia.” Heliyon 8, no. 10: e10791. 10.1016/j.heliyon.2022.e10791. 36203897 PMC9529577

[fsn371684-bib-0002] Ba, D. M. , P. Ssentongo , X. Gao , et al. 2022. “Prevalence and Determinants of Meeting Minimum Dietary Diversity Among Children Aged 6‐23 Months in Three Sub‐Saharan African Countries: The Demographic and Health Surveys, 2019‐2020.” Frontiers in Public Health 10: 846049. 10.3389/fpubh.2022.846049. 36081474 PMC9445207

[fsn371684-bib-0003] Bandalos, D. L. 2014. “Relative Performance of Categorical Diagonally Weighted Least Squares and Robust Maximum Likelihood Estimation.” Structural Equation Modeling: A Multidisciplinary Journal 21, no. 1: 102–116. 10.1080/10705511.2014.859510.

[fsn371684-bib-0004] Besnier, E. , K. Thomson , D. Stonkute , et al. 2019. “Which Public Health Interventions Are Effective in Reducing Morbidity, Mortality and Health Inequalities From Infectious Diseases Amongst Children in Low‐Income and Middle‐Income Countries (LMICs): Protocol for an Umbrella Review.” BMJ Open 9, no. 12: e032981. 10.1136/bmjopen-2019-032981. PMC6937061 31888932

[fsn371684-bib-0005] Black, M. M. , C. K. Lutter , and A. C. B. Trude . 2020. “All Children Surviving and Thriving: Re‐Envisioning UNICEF'S Conceptual Framework of Malnutrition.” Lancet. Global Health 8, no. 6: e766–e767. 10.1016/S2214-109X(20)30122-4. 32446344

[fsn371684-bib-0006] Black, R. E. , S. S. Morris , and J. Bryce . 2003. “Where and Why Are 10 Million Children Dying Every Year?” Lancet 361, no. 9376: 2226–2234. 10.1016/S0140-6736(03)13779-8. 12842379

[fsn371684-bib-0007] Black, R. E. , C. G. Victora , S. P. Walker , et al. 2013. “Maternal and Child Undernutrition and Overweight in Low‐Income and Middle‐Income Countries.” Lancet 382, no. 9890: 427–451. 10.1016/S0140-6736(13)60937-X. 23746772

[fsn371684-bib-0008] Choudhury, N. , M. J. Raihan , S. Sultana , et al. 2017. “Determinants of Age‐Specific Undernutrition in Children Aged Less Than 2 Years‐The Bangladesh Context.” Maternal & Child Nutrition 13, no. 3: e12362. 10.1111/mcn.12362. 27731545 PMC6865922

[fsn371684-bib-0009] Eunice, M. J. , W. L. Cheah , and P. Y. Lee . 2014. “Factors Influencing Malnutrition Among Young Children in a Rural Community of Sarawak.” Malaysian Journal of Nutrition 20, no. 2: 145–164.

[fsn371684-bib-0010] Fufa, D. A. , and T. D. Laloto . 2021. “Factors Associated With Undernutrition Among Children Aged Between 6–36 Months in Semien Bench District, Ethiopia.” Heliyon 7, no. 5: e07072. 10.1016/j.heliyon.2021.e07072. 34041409 PMC8141868

[fsn371684-bib-0011] Gelli, A. , A. Margolies , M. Santacroce , et al. 2018. “Using a Community‐Based Early Childhood Development Center as a Platform to Promote Production and Consumption Diversity Increases Children's Dietary Intake and Reduces Stunting in Malawi: A Cluster‐Randomized Trial.” Journal of Nutrition 148, no. 10: 1587–1597. 10.1093/jn/nxy148. 30204916 PMC6168702

[fsn371684-bib-0012] González‐Fernández, D. , O. Muralidharan , P. A. Neves , and Z. A. Bhutta . 2024. “Associations of Maternal Nutritional Status and Supplementation With Fetal, Newborn, and Infant Outcomes in Low‐Income and Middle‐Income Settings: An Overview of Reviews.” Nutrients 16, no. 21: 3725. 10.3390/nu16213725. 39519557 PMC11547697

[fsn371684-bib-0013] Goudet, S. , P. Griffths , and B. A. Bogin . 2011. “Mother's Body Mass Index as a Predictor of Infant's Nutritional Status in the Post‐Emergency Phase of a Flood.” Disasters 35, no. 4: 701–719. 10.1111/j.1467-7717.2011.01238.x. 21913932

[fsn371684-bib-0014] Hasanah, A. , B. Kharisma , S. S. Remi , A. M. Adam , and A. Y. M. Siregar . 2024. “Food Diversity: Its Relation to Children's Health and Consequent Economic Burden.” BMC Public Health 24, no. 1: 1155. 10.1186/s12889-024-18530-w. 38658917 PMC11044496

[fsn371684-bib-0015] Herforth, A. , and J. Harris . 2014. “Understanding and Applying Primary Pathways and Principles.” Brief #1. Improving Nutrition through Agriculture Technical Brief Series. Arlington, VA: USAID/Strengthening Partnerships, Results, and Innovations in Nutrition Globally (SPRING) Project.

[fsn371684-bib-0016] Katoch, O. R. 2022. “Determinants of Malnutrition Among Children: A Systematic Review.” Nutrition 96: 111565. 10.1016/j.nut.2021.111565. 35066367

[fsn371684-bib-0017] Kuchenbecker, J. , A. Reinbott , B. Mtimuni , M. B. Krawinkel , and I. Jordan . 2017. “Nutrition Education Improves Dietary Diversity of Children 6‐23 Months at Community‐Level: Results From a Cluster Randomized Controlled Trial in Malawi.” PLoS One 12, no. 4: e0175216. 10.1371/journal.pone.0175216. 28426678 PMC5398527

[fsn371684-bib-0018] Liu, L. , H. L. Johnson , S. Cousens , et al. 2012. “Global, Regional, and National Causes of Child Mortality: An Updated Systematic Analysis for 2010 With Time Trends Since 2000.” Lancet 379, no. 9832: 2151–2161. 10.1016/S0140-6736(12)60560-1. 22579125

[fsn371684-bib-0019] Lye, C. W. , S. Sivasampu , T. Mahmudiono , and H. A. Majid . 2023. “A Systematic Review of the Relationship Between Household Food Insecurity and Childhood Undernutrition.” Journal of Public Health (Oxford, England) 45, no. 4: e677–e691. 10.1093/pubmed/fdad070. 37291061

[fsn371684-bib-0020] Ministry of Health (MoH) , Uganda Bureau of Statistics (UBOS) , Office of the Prime Minister (OPM) , and The Office of the United Nations High Commissioner for Refugees (UNHCR) . 2020. “Food Security and Nutrition Assessment in Refugee Settlements and Kampala, December 2020.”

[fsn371684-bib-0021] Morrissey, K. , T. Reynolds , D. Tobin , and C. Isbell . 2023. “Market Engagement, Crop Diversity, Dietary Diversity, and Food Security: Evidence From Small‐Scale Agricultural Households in Uganda.” Food Security 16: e014112. 10.1007/s12571-023-01411-2.

[fsn371684-bib-0022] Motbainor, A. , A. Worku , and A. Kumie . 2015. “Stunting Is Associated With Food Diversity While Wasting With Food Insecurity Among Underfive Children in East and West Gojjam Zones of Amhara Region, Ethiopia.” PLoS One 10, no. 8: e0133542. 10.1371/journal.pone.0133542. 26285047 PMC4540277

[fsn371684-bib-0023] Mwesigye, F. , and M. Barungi . 2021. “Land Tenure Insecurity, Fragmentation and Crop Choice.” African Economic Research Consortium.

[fsn371684-bib-0024] Nassur, A. M. , O. Daanouni , G. Luc , et al. 2022. “Factors Associated With Acute Malnutrition Among Children Aged 6‐59 Months in Haiti, Burkina Faso and Madagascar: A Pooled Analysis.” PLoS One 17, no. 12: e0278980. 10.1371/journal.pone.0278980. 36508472 PMC9744306

[fsn371684-bib-0025] Negash, C. , S. J. Whiting , C. J. Henry , T. Belachew , and T. G. Hailemariam . 2015. “Association Between Maternal and Child Nutritional Status in Hula, Rural Southern Ethiopia: A Cross Sectional Study.” PLoS One 10, no. 11: e0142301. 10.1371/journal.pone.0142301. 26588687 PMC4654505

[fsn371684-bib-0026] Odei Obeng‐Amoako, G. A. , C. A. S. Karamagi , J. Nangendo , et al. 2021. “Factors Associated With Concurrent Wasting and Stunting Among Children 6‐59 Months in Karamoja, Uganda.” Maternal & Child Nutrition 17, no. 1: e13074. 10.1111/mcn.13074. 32830434 PMC7729532

[fsn371684-bib-0027] Osei, A. , P. Pandey , J. Nielsen , et al. 2017. “Combining Home Garden, Poultry, and Nutrition Education Program Targeted to Families With Young Children Improved Anemia Among Children and Anemia and Underweight Among Nonpregnant Women in Nepal.” Food and Nutrition Bulletin 38, no. 1: 49–64. 10.1177/0379572116676427. 27837036

[fsn371684-bib-0028] Pandey, V. L. , S. Mahendra Dev , and U. Jayachandran . 2016. “Impact of Agricultural Interventions on the Nutritional Status in South Asia: A Review.” Food Policy 62: 28–40. 10.1016/j.foodpol.2016.05.002. 27478297 PMC4952527

[fsn371684-bib-0029] Ruel, M. T. , H. Alderman , and Maternal and Child Nutrition Study Group . 2013. “Nutrition‐Sensitive Interventions and Programmes: How Can They Help to Accelerate Progress in Improving Maternal and Child Nutrition?” Lancet 382, no. 9891: 536–551. 10.1016/S0140-6736(13)60843-0. 23746780

[fsn371684-bib-0030] UNICEF . 1990. “Strategy for Improved Nutrition of Children and Women in Developing Countries.” 10.1007/BF02810402 1937618

[fsn371684-bib-0031] Victora, C. G. , L. Adair , C. Fall , et al. 2008. “Maternal and Child Undernutrition: Consequences for Adult Health and Human Capital.” Lancet 371, no. 9609: 340–357. 10.1016/S0140-6736(07)61692-4. 18206223 PMC2258311

[fsn371684-bib-0032] von Grebmer, K. , J. Bernstein , W. Geza , et al. 2023. 2023 Global Hunger Index: The Power of Youth in Shaping Food Systems. Welthungerhilfe (WHH); Concern Worldwide.

[fsn371684-bib-0033] World Health Organization . 2023. Levels and Trends in Child Malnutrition Child Malnutrition: UNICEF/WHO/World Bank Group Joint Child Malnutrition Estimates. World Health Organization.

[fsn371684-bib-0034] Zeinalabedini, M. , B. Zamani , E. Nasli‐Esfahani , and L. Azadbakht . 2023. “A Systematic Review and Meta‐Analysis of the Association of Dietary Diversity With Undernutrition in School‐Aged Children.” BMC Pediatrics 23, no. 1: 269. 10.1186/s12887-023-04032-y. 37246212 PMC10226245

